# The role of courtship song in female mate choice in South American Cactophilic *Drosophila*

**DOI:** 10.1371/journal.pone.0176119

**Published:** 2017-05-03

**Authors:** Patricia P. Iglesias, Esteban Hasson

**Affiliations:** 1 Departamento de Ecología, Genética y Evolución, Facultad de Ciencias Exactas y Naturales, Universidad de Buenos Aires, Ciudad Autónoma de Buenos Aires, Argentina; 2 Instituto de Ecología, Genética y Evolución de Buenos Aires, Consejo Nacional de Investigaciones Científicas y Técnicas, Ciudad Autónoma de Buenos Aires, Argentina; CNRS, FRANCE

## Abstract

Courtship songs have undergone a spectacular diversification in the *Drosophila buzzatii* cluster. Accordingly, it has been suggested that sexual selection has played a significant role in promoting rapid diversification, reproductive isolation and speciation. However, there is no direct evidence (*i*.*e*., song playback experiments with wingless males) supporting this tenet. Moreover, several studies have showed that the courtship song in the genus *Drosophila* is not always used in female mate choice decisions, nor plays the same role when it is taken into account. In this vein, we use an approach that combines manipulative and playback experiments to explore the importance and the role of courtship song in female mate choice in four species of the *D*. *buzzatii* cluster and one species of the closely related *D*. *martensis* cluster for outgroup comparison. We also investigate the importance of courtship song in sexual isolation in sympatry between the only semi-cosmopolitan species, *D*. *buzzatii*, and the other species of the *D*. *buzzatii* cluster. Our study revealed that the courtship song is used by females of the *D*. *buzzatii* cluster as a criterion for male acceptance or influences the speed with which males are chosen. In contrast, we showed that this characteristic is not shared by *D*. *venezolana*, the representative species of the *D*. *martensis* cluster. We also found that the studied species of the *D*. *buzzatii* cluster differ in the role that conspecific and heterospecific songs have in female mate choice and in sexual isolation. Our findings support the hypothesis that divergence in female preferences for courtship songs has played a significant role in promoting rapid diversification and reproductive isolation in the *D*. *buzzatii* cluster. However, evidence from *D*. *venezolana* suggests that the use of the courtship song in female mate choice is not a conserved feature in the *D*. *buzzatii* complex.

## Introduction

Males display multiple signals of different sensory modalities during courtship and most of these signals have undergone spectacular diversification even in groups of closely related species [1, 2]. However, it is not clear whether all of these signals are important in mate choice, or whether females emphasize one type of signal over others [3–5].

In flies of the genus *Drosophila*, males produce an innate complex courtship behavior in which acoustic and chemical signals are thought to be the more conspicuous targets of female mate choice [6]. Among these signals, the courtship song produced by wing vibrations has been the most extensively studied (*e*.*g*., [7–14]). Previous works evaluating the role of mating song in sexual selection and sexual isolation showed wide variation across species. For instance, it has been suggested that the courtship song of *D*. *heteroneura* males (*D*. *planitibia* group) does not influence male mating success and, therefore, cannot be considered a sexual signal [15]. On the other hand, females of *D*. *montana* (*D*. *virilis* group) reject the courting male if they do not hear the song [16]. In some species of the *D*. *melanogaster* complex, courtship song does not seem to be necessary for mating, although it decreases the time to copulation [17, 18]. In addition, there is evidence that song characters do not need to be species-specific since heterospecific songs can also stimulate female sexual behavior in some species of the *D*. *melanogaster* complex [17, 18]. Nevertheless, females of *D*. *sechellia* (*D*. *simulans* complex) and *D*. *biauraria* (*D*. *auraria* complex) reject males when song characters are heterospecific [19, 20]. Overall, these studies show that although courtship songs vary extensively across species, it does not mean that they always play an important role in female mate choice or mate recognition [17].

The *D*. *buzzatii* complex consists of three species clusters, namely *D*. *buzzatii*, *D*. *martensis* and *D*. *stalkeri*. The distribution area comprises Argentina, Brazil, Bolivia and Paraguay (*D*. *buzzatii* cluster), Colombia and Venezuela (*D*. *martensis* cluster), and the Caribbean Islands and Florida (*D*. *stalkeri* cluster) [21]. Flies of the *D*. *buzzatii* complex inhabit arid and/or semiarid environments where they breed and feed on fermenting cacti, either prickly pears (genus *Opuntia*) or columnar cacti [21, 22]. Some species are host specialists, whereas others use predominantly one type of host (primary host) and less frequently alternative hosts (secondary hosts), indicating some degree of niche overlap. The evolutionary relationships within the *D*. *buzzatii* complex are poorly known since nodes have low support in all phylogenetic hypotheses proposed. Chromosomal [21] and molecular sequence data (including the nuclear gene Xdh and the mitochondrial genes COI, COII and COIII) [23] suggest that the *D*. *stalkeri* cluster is the oldest lineage and is paraphyletic with respect to the *D*. *buzzatii* and *D*. *martensis* clusters that are reciprocally monophyletic sister clades. However, recent work based on more extensive molecular data (four mitochondrial and six nuclear markers) recovered *D*. *buzzatii* and *D*. *stalkeri* clusters as sister clades [22].

Recent studies of courtship songs and cuticular hydrocarbons (CHC) in the *D*. *buzzatii* cluster, revealed rapid evolution of male courtship songs [24] and a certain degree of conservatism in CHC profiles [25]. In fact, despite quantitative differences among species, CHC profiles can be predicted by species ancestry. Therefore, it was suggested that courtship songs may encode information that mediates mating discrimination between species [24]. However, there is no direct evidence (*i*.*e*., song playback experiments with wingless males) supporting this tenet.

The evolution of male courtship songs in cactophilic flies of the *D*. *buzzatii* cluster is characterized by a pattern of losses and/or elaboration of their components [24]. Songs are composed of low-frequency, polycyclic pulses arranged into pulse trains that have different structural and temporal features [24]. Thus, some species like *D*. *borborema* and *D*. *gouveai* perform one type of song whereas others like *D*. *antonietae*, *D*. *buzzatii*, *D*. *koepferae*, *D*. *serido* and *D*. *seriema* perform two. Moreover, the courtship song of *D*. *buzzatii* males is the only one with a bimodal distribution of interpulse intervals (IPIs) in the primary or A song ([11]; P.P. Iglesias pers obs). Surprisingly, Oliveira *et al*. [24] did not report this tendency to alternate pulses with long and short IPI in the primary or A song of *D*. *buzzatii* males. However, a bimodal distribution of IPIs in the primary song can be detected by measuring the IPIs in the sonogram of Fig 20C of Oliveira *et al*. [24] (see Discussion for more details). In addition, among species with two types of songs, only *D*. *buzzatii* males produced significantly different primary and secondary songs, *i*.*e*., short IPI in the primary song and long IPI in the secondary song ([24]; P.P. Iglesias pers obs). IPI differences are interesting since insects usually have low spectral resolution and it is the temporal component that carries most information [26]. The importance of IPI in species recognition has been demonstrated in several species of *Drosophila* (reviewed in [27]).

**Fig 1:**
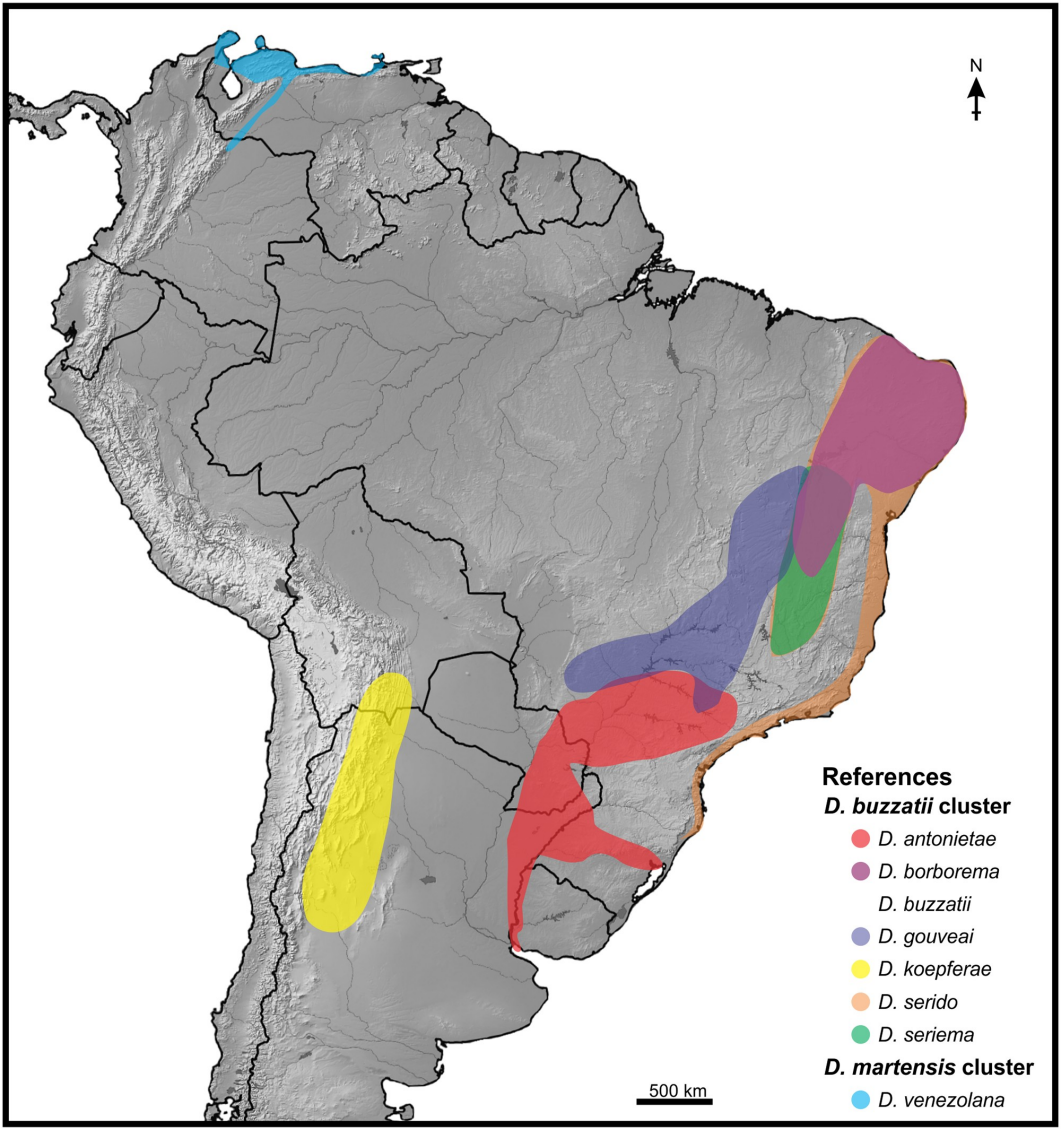
Geographic distribution of the seven species of the *D*. *buzzatii* cluster and *D*. *venezolana* (*D*. *martensis* cluster). The distribution area of *D*. *buzzatii* overlaps with all in-group species; therefore, it is not marked on the map. Sources: Cerda & Fontdevila [31], Manfrin & Sene [28], Oliveira *et al*. [25], and P.P. Iglesias, J. Hurtado, and E. Hasson, unpublished field data. The map was created using SimpleMappr http://www.simplemappr.net/ and edited in CorelDRAW X7.

Besides having a divergent courtship song, *D*. *buzzatii* also differs from the other six species of the cluster (the so-called “*D*. *serido* sibling set”; [28]) in ecological, morphological and biogeographic characteristics. First, it is the only species that prefers decaying tissues of prickly pears of the genus *Opuntia* as primary hosts, although it can also use columnar cacti as secondary hosts. Second, It has the most divergent aedeagus morphology (male genitalia), in both size and shape, as compared to the other species. Finally, it is the only widespread species that co-occurs with all other species of the cluster in different areas of its vast geographic range in South America ([28, 29]; Fig 1). These features make *D*. *buzzatii* an interesting target for evolutionary studies of traits involved in precopulatory sexual isolation because the strengthening of the mechanisms involved in mate discrimination can avoid the potential harmful hybridization [30]. In this sense, the courtship song of *D*. *buzzatii* males is a salient precopulatory sexual trait since it is complex enough to be easily discriminated by females of the species of the *D*. *serido* sibling set and to be easily recognized by conspecific females.

In the present study we use an approach that combines manipulative and playback experiments to explore the importance and the role of courtship song in female mate choice in four species of the *D*. *buzzatii* cluster and one species of the closely related *D*. *martensis* cluster for outgroup comparison. In addition, we focus on the importance of courtship song in sexual isolation in sympatry between *D*. *buzzatii* and the other species of the *D*. *buzzatii* cluster. We studied female mate choice in terms of variation in two aspects: which acoustic conditions favor male acceptance and how fast are males accepted in each acoustic condition.

## Materials and methods

### *Drosophila* stocks

We used 13 isofemale lines of four species of the *D*. *buzzatii* cluster namely: *D*. *antonietae* (4 lines), *D*. *borborema* (1), *D*. *buzzatii* (4), and *D*. *koepferae* (4). Isofemales lines of *D*. *buzzatii* and *D*. *koepferae* were originally founded with the progeny of wild inseminated females collected in the San Agustín del Valle Fértil natural reserve (San Juan Province, western Argentina) and *D*. *antonietae* lines derive from wild inseminated females collected in Isla Martín García (Buenos Aires Province, east-central Argentina) where it coexists with *D*. *buzzatii* (J. Hurtado, P.P. Iglesias & E. Hasson, unpublished field data). These lines were collected in March 2012 and maintained for about 18 months before the experiments. The *D*. *borborema* line was obtained from the *Drosophila* Species Stock Center (Stock Number: 15081–1281.01; University of California, San Diego, USA) and derives from the progeny of a wild inseminated female collected in 1974 in Morro do Chapéu (Bahia state, Brazil) where it also coexists with *D*. *buzzatii* [32]. We also included an isofemale line of *D*. *venezolana* of the *D*. *martensis* cluster for outgroup comparison. This species is also a cactophilic fly that is distributed in northern South America and is allopatric to all *D*. *buzzatii* cluster species ([21]; Fig 1). This line was kindly provided by Antonio Fontdevila and derives from the progeny of a wild inseminated female collected in 1984 in Isla Los Roques (Venezuela) [31]).

### Experimental conditions

**Fig 2:**
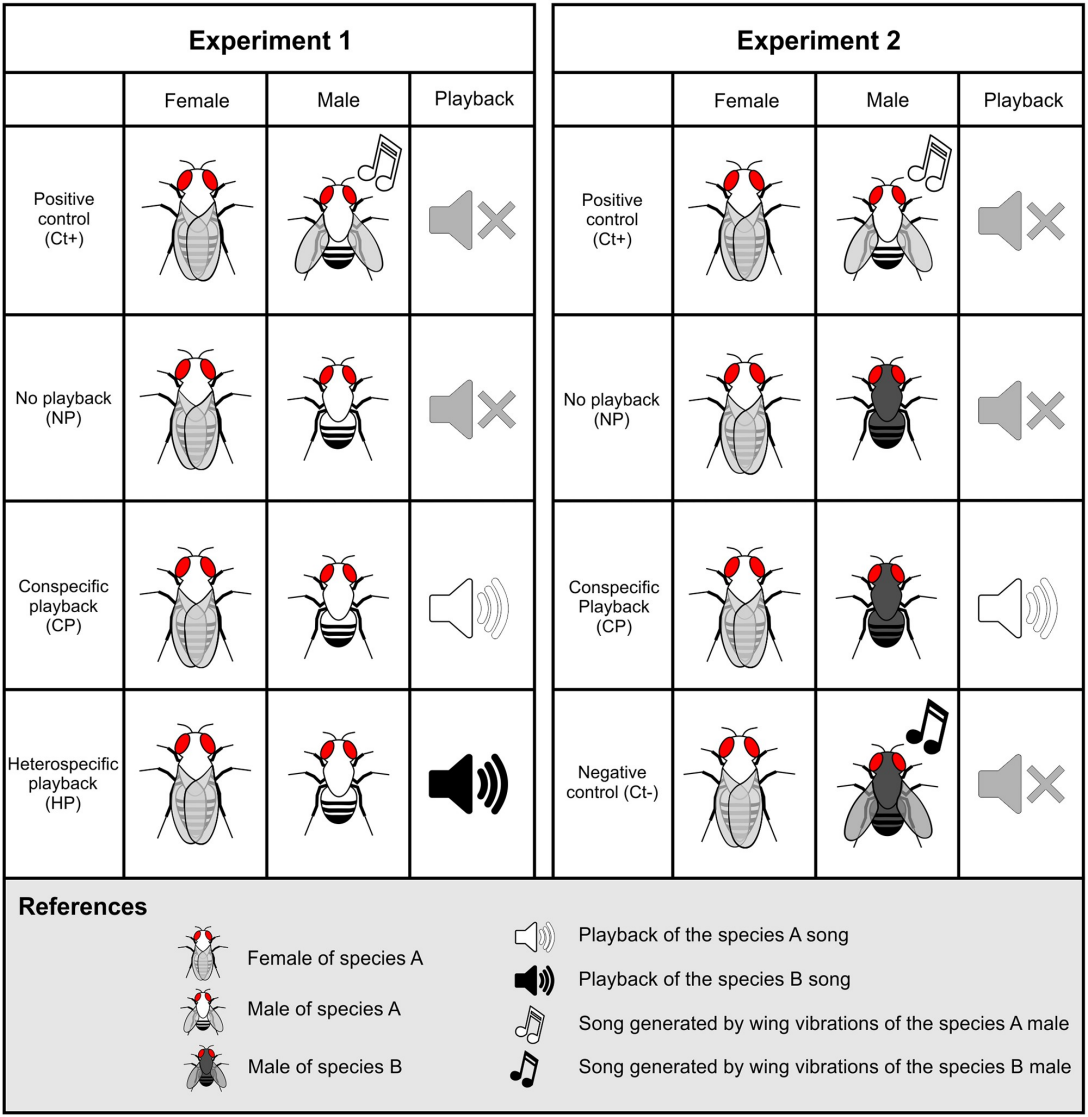
An schematic representation of the acoustic conditions employed in the experiments. An example showing how female mate choice for a hypothetical species “A” was studied across the different acoustic conditions, and using conspecific and heterospecific males and songs in each experiment.

Two types of no choice experiments were performed (Fig 2). In Experiment 1 we investigated the importance and the role of courtship song in female mate choice when unmanipulated females were exposed to conspecific males without wings (*i*.*e*., without the structures involved in the emission of songs) in three different acoustic conditions: with no playback (NP), with conspecific song playback (CP), and with heterospecific song playback (HP). We also included a positive control (Ct+) that consisted of pairs of conspecific winged males and females. Since the song of *D*. *buzzatii* is the most divergent and all females of the *D*. *buzzatii* cluster derive from collections in localities where they coexist with *D*. *buzzatii*, we used playbacks of *D*. *buzzatii* song as heterospecific playbacks in the HP condition. In trials involving *D*. *buzzatii* females, we played back *D*. *koepferae* songs in the HP condition because lines of both species derive from the same locality.

Experiment 2 aimed to explore the role of courtship song in sexual isolation between *D*. *buzzatii* and the other species of the cluster included in this study (Fig 2). To this end, we confronted unmanipulated females of *D*. *borborema*, *D*. *antonietae* or *D*. *koepferae* with wingless *D*. *buzzatii* males in two different acoustic conditions: with no playback (NP) and with conspecific song playback (CP). In this experiment we included two types of controls: a positive control (Ct+) identical to the control group of Experiment 1, using winged conspecific males, and a negative control (Ct-), using winged heterospecific *D*. *buzzatii* males, to corroborate the existence of precopulatory sexual isolation between the species involved. As in Experiment 1, in trials involving *D*. *buzzatii* females, we use wingless *D*. *koepferae* males as heterospecifics because lines of both species come from an area where both species coexist.

Experiments were carried out between 8:00 AM and 11:00 AM when flies showed highest mating activity, at 25 ± 1°C. Flies were sexed under light CO_2_ anesthesia within 6 h of eclosion, when wings were already extended. At this moment, since wingless males were required for some assays, both wings of some males were removed with a pair of microsurgical scissors from the base of the wing whereas other males remained unmanipulated (winged males). Thus, both winged and wingless males were exposed to light CO_2_ anesthesia for the same time span. Both types of males and females were maintained separately for 5 to 7 days in food vials until their use in experiments.

### Behavioral variables measured

To evaluate the effects of the different acoustic conditions, we measured two aspects of female mate choice: mate acceptance (MA) and copulation latency (CL). Mate acceptance was computed as a binary measure, 1 when copulation occurred and 0 otherwise. Copulation latency was measured as the time elapsed since the male began to follow the female until copulation. The observation period was limited to 10 minutes (= min) since female latency time started to be measured.

Measuring mating behavioral variables since males initiated courtship excludes variation due to male preferences, especially in Experiment 2 in which we assessed heterospecific pairs. Therefore, CL can be compared between conditions involving conspecific males (Ct+) and heterospecific males (Ct-, NP and CP) without bias. Also, it is important to take into account that *D*. *buzzatii* males (or *D*. *koepferae* when appropiate) are very promiscuous and, once courtship begins, they persist even when the female kicks, raises and lowers her abdomen, or move around.

We used copulation duration to define MA in heterospecific pairs on Experiment 2. Since copulation duration could be shortened in heterospecific pairs by processes involving postcopulatory preferences or discrimination [33–35], considering only prolonged copulations would underestimate the female response to the male precopulatory behavior. Accordingly, we recorded as acceptance when the female remained still for at least 10 seconds (= s) after the pair were in-copula without exhibiting rejection behavior (*i*.*e*., kicking or moving around), which minimized pseudo-copulations. We recorded copulation duration in all couples in Experiment 2 (see Table 1) and our criterion is discussed.

**Table 1:** Copulation duration (in s) registered in pairs of Experiment 2 using winged conspecific (Ct+) or heterospecific (Ct-) males and wingless heterospecific males in two different acoustic conditions (NP, CP).

	*D*. *antonietae*			*D*. *borborema*			*D*. *buzzatii*			*D*. *koepferae*		
	$\bar{\text{x}}$	Min	Max	$\bar{\text{x}}$	Min	Max	$\bar{\text{x}}$	Min	Max	$\bar{\text{x}}$	Min	Max
Ct+	257	191	1559	190	105	290	119	78	229	202	141	307
NP	—	—	—	—	—	—	—	—	—	—	—	—
CP	356	89	1974	48	21	187	68	37	148	—	—	—
Ct-	—	—	—	—	—	—	—	—	—	—	—	—

Ct+ = positive control (using winged conspecific males); NP = wingless heterospecific males with no playback; CP = wingless heterospecific males with conspecific playback; Ct- = negative control (using winged heterospecific males). $\bar{\text{x}}$ = mean; Min = minimum duration; Max = maximum duration.

We assessed 30 pairs for each combination of species, isofemale line and acoustic condition in each experiment, except in the case of *D*. *borborema* in Experiment 2 in which two replicates were discarded since flies took too long to initiate courtship. All isofemale lines were used in Experiment 1; however, the long time required by the males to start courting in heterospecific pairs (whose effect our experimental design is intended to rule out) and our interest in recording the duration of copulation, prevented us to evaluate a large number of pairs. Thus, one line per species was used in Experiment 2.

### Song recording and playback

**Fig 3:**
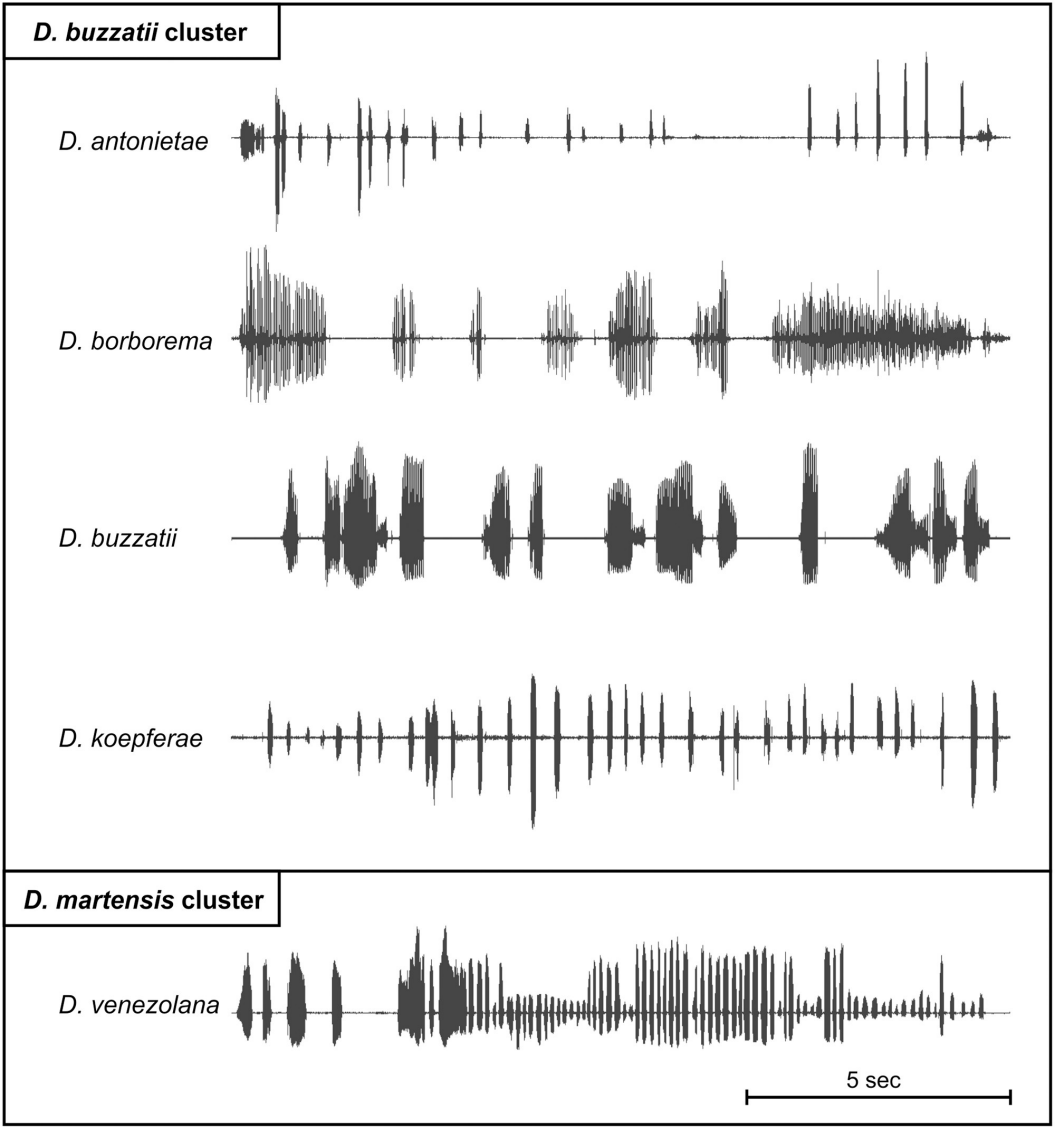
Representative sonograms of the courtship songs of the five species recorded for playback experiments.

Song playbacks used in this study were recorded individually from ten males for each combination of isofemale line and species with a SONY ICD-SX712 in an uncompressed Linear PCM wav file, and using a sampling rate of 44.1 kHz and 16-bit precision. Recordings, in the same channel, were carried out using the SONY ICD-SX712 built-in microphone in an acoustically isolated room at 25±0.3°C (measured with a digital thermometer) controlled by a heating/cooling system. Mating arenas consisted of polymethyl methacrylate cylinders 0.5 mm height and 1.2 mm in internal diameter, the same diameter as the microphone grid. The floor of the chamber was the microphone itself and had a foam cap in the opposite extreme where flies were introduced. Males were exposed to females whose wings had been removed to avoid interferences during recording and songs were recorded until copulation. Before being used in playback experiments, songs were edited by eliminating the period of time elapsed before the male began to sing. We employed the software Sound Forge 9.0 (Sony Creative Software Inc., 2007) for song editing. Representative sonograms of the courtship songs of the five species recorded in this work are illustrated in Fig 3.

For playback assays we used the same SONY ICD-SX712 equipment coupled to SONY MDR-E708 speakers. In these assays, mating arenas were 1.2 mm height and we replaced the microphone by a speaker. The ten songs recorded for each isofemale line were randomly used in each test by line (each song was used three times in each acoustic condition by line) and the playback started when the male began to follow the female. In HP condition, we chose 10 random songs of each species used as heterospecifics (*i*.*e*., *D*. *buzzatii* or *D*. *koepferae* when appropriate) and we used it in the manner described above.

The whole set of replicates by species was randomized into different assay sessions to avoid confounding effects.

#### Statistical analysis

Since both variables measured in this study describe two different aspects of mate choice [36], “what” is preferred (mate acceptance) and “by how much” (mating latency time), we decided to analyze them separately.

All models included acoustic condition as a fixed factor, and, for *D*. *antonietae*, *D*. *buzzatii* and *D*. *koepferae* in Experiment 1, isofemale line was also included as a random factor (only in these cases we used mixed models). For mixed models, we used a random slope model, which allows for the relationship between the acoustic conditions and the female responses to be different for each isofemale line [37]. Since this model failed to converge in *D*. *koepferae*, we fitted a random slope with fixed intercept model in this species.

Therefore, to test if mate acceptance differed among acoustic conditions, a generalized linear model (GLM) or a generalized linear mixed model (GLMM; *lme4* package [38]) both with a binomial distribution of errors and a logit link was fitted for each species as appropriate. Tukey contrasts failed when we compared some acoustic conditions in which all females mated (mate acceptance status = 1) versus acoustic conditions in which all females refused to mate (mate acceptance status = 0); therefore, we changed the mate acceptance status of a single individual in some ‘acoustic condition × isofemale line’ groups.

To investigate differences in copulation latency among acoustic conditions with more than 50 percent of mate acceptance, we used a linear model (LM) or a linear mixed model (LMM; *lme4* package). The time variable was log-transformed to better meet the assumptions of homoscedasticity and normally distributed errors. Type III sums-of-squares (SS) was used to account for the unbalanced design ('car' package [39]).

Tukey single-step tests were carried out using the 'multcomp' package [40]. All statistical analyses were run in R software version 3.2.0 [41]. We used Kaplan–Meier plots (survival package, [42]) to graphically summarize all experimental results.

## Results

### Experiment 1: Significance of the courtship song in conspecific pairs

**Fig 4:**
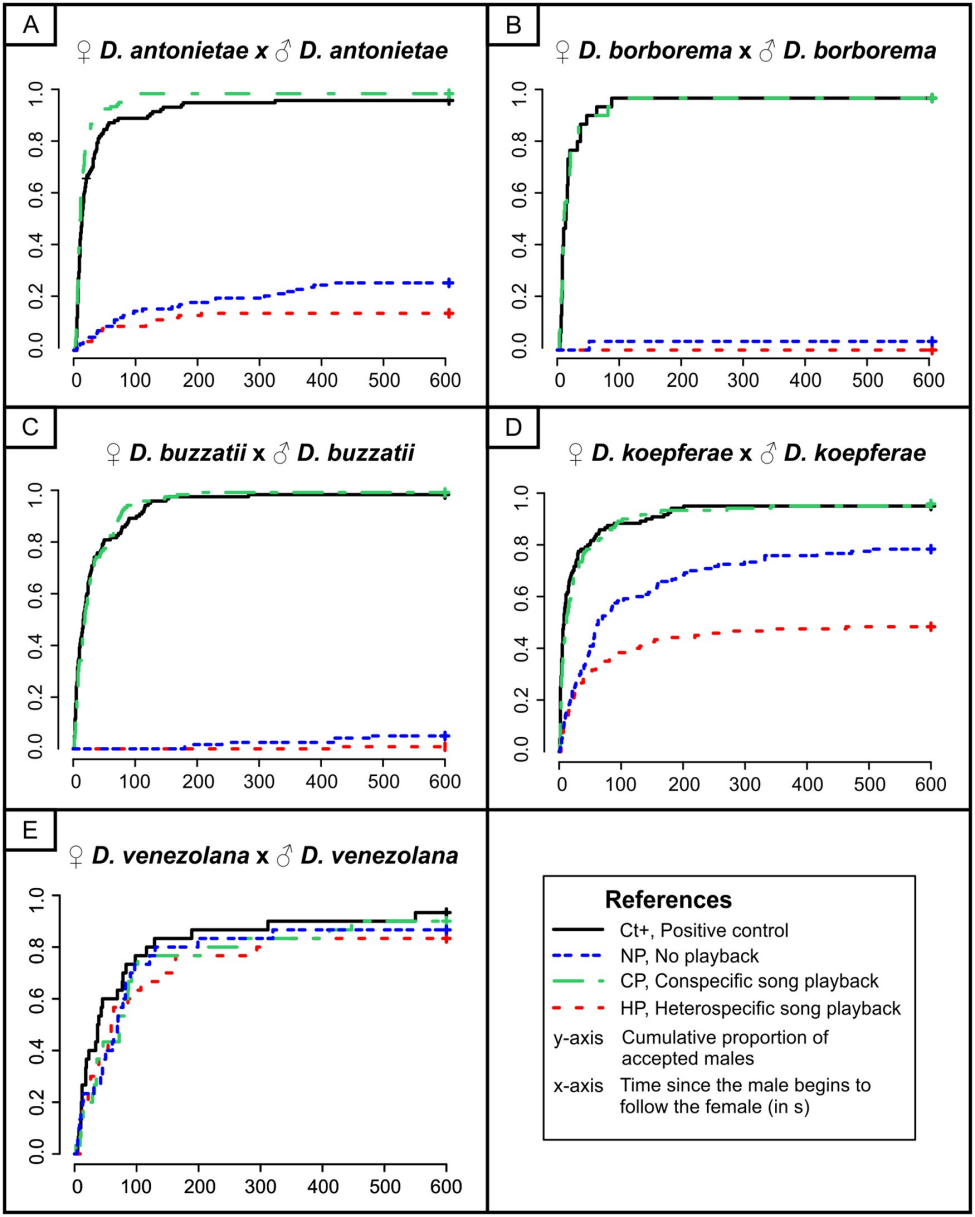
Interspecific variation in female response under different acoustic conditions in conspecific pairs. Effect of different acoustic conditions on mate acceptance and mating latency in four species of the *D*. *buzzatii* cluster and *D*. *venezolana* (*D*. *martensis* cluster). Winged males were used in Ct+ and wingless males in NP, CP and HP conditions. Data obtained for the isofemale lines of *D*. *antonietae*, *D*. *buzzatii* and *D*. *koepferae* are pooled in this figure.

A total of 1320 couples were assessed to evaluate the significance of acoustic communication in conspecific pairs in the four species of the *D*. *buzzatii* cluster and *D*. *venezolana*. The results of the effects of the acoustic conditions in the five species assessed are summarized in Fig 4. All statistical pairwise comparisons and associated adjusted p-values can be found in S1 and S2 Tables for variables MA and CL, respectively.

The analysis of the data revealed a significant effect of the acoustic stimulus on mate acceptance (MA) in all species of the *D*. *buzzatii* cluster assayed (*D*. *antonietae*, *D*. *borborema*, *D*. *buzzatii*, and *D*. *koepferae*). In effect, MA was significantly reduced (relative to controls) when wingless males court in the absence of song playback (Fig 4A–4D, NP condition; S1 Table). This implies that wings are important for male mating success, but does not establish the importance of the courtship song. Although at least one female accepted the male in the absence of playback in all species, the reduction in MA was much more marked in *D*. *borborema* (Fig 4B) and *D*. *buzzatii* (Fig 4C), intermediate in *D*. *antonietae* (Fig 4A), whereas *D*. *koepferae* females were the least affected by the absence of male wings (Fig 4D). The high acceptance of wingless males in the absence of playback by *D*. *koepferae* females was accompanied by a significant increase in the mean time to achieve copulation (NP: 87±106 s vs. Ct+: 25±43 s, Tukey test: *Z* = 6.77, *P* < 0.001). These results show that there are interspecific differences in relation to the effect that the absence of male wings exerts on female mate choice. The presence or absence of male wings determined the acceptance or rejection of males in most *D*. *antonietae*, *D*. *borborema* and *D*. *buzzatii* females, whereas male wings in *D*. *koepferae* primarily reduced the necessary time to be accepted by females.

To determine the role of courtship song on female mate choice, we tested wingless males either with the conspecific or a heterospecific song playback.

Similar to the results obtained in assays without any playback (NP condition), MA was significantly reduced (relative to the Ct+ group) when wingless males courted with the heterospecific song playback (Fig 4A–4D; HP condition; S1 Table). However, the conspecific song playback (CP condition) restored female mating behavior in terms of both mate acceptance and copulation latency in all species (Fig 4A–4D; S1 and S2 Tables). These results indicate that conspecific song playback is enough to compensate for the absence of wings. Therefore, the reduction in MA and the increased in CL in assays involving wingless males with no playback can be explained only by the absence of the conspecific song. Moreover, no significant differences were found between heterospecific song playback (HP) and no playback (NP) conditions in *D*. *antonietae*, *D*. *borborema* and *D*. *buzzatii* (Fig 4A–4C; S1 Table). These results suggest that heterospecific song playback neither stimulates nor reduces MA in *D*. *antonietae*, *D*. *borborema* and *D*. *buzzatii*. However, the picture was quite different in *D*. *koepferae*, since the heterospecific song playback had a negative effect on MA (HP: 0.48; Fig 4D, HP condition) as compared to the assays without playback (NP: 0.79; Tukey test: Z = 4.77, P <0.001). Consequently, only in the case of *D*. *koepferae* we found evidence that support a reduction in MA by the heterospecific song playback.

Finally, to explore whether the patterns observed in species of the *D*. *buzzatii* cluster regarding the importance of acoustic stimulus in female mate choice are conserved in the *D*. *buzzatii* complex or, on the contrary, there is evidence of greater diversity, we included *D*. *venezolana*, a species of the closely related *D*. *martensis* cluster. Our results revealed that the different acoustic conditions assessed in *D*. *venezolana* (Fig 4E) did not differ in MA (GLM: $\chi_{3}^{2}$ = 1.66, *P* = 0.65) nor in CL (LM: *F*_3,102_ = 0.46, *P* = 0.71). These results show not only that *D*. *venezolana* females do not require the conspecific song or wings to accept males, but also that the heterospecific song (even though *D*. *buzzatii* is largely allopatric to *D*. *venezolana*) does not affect female mate choice.

### Experiment 2: Significance of the courtship song in sexual isolation

**Fig 5:**
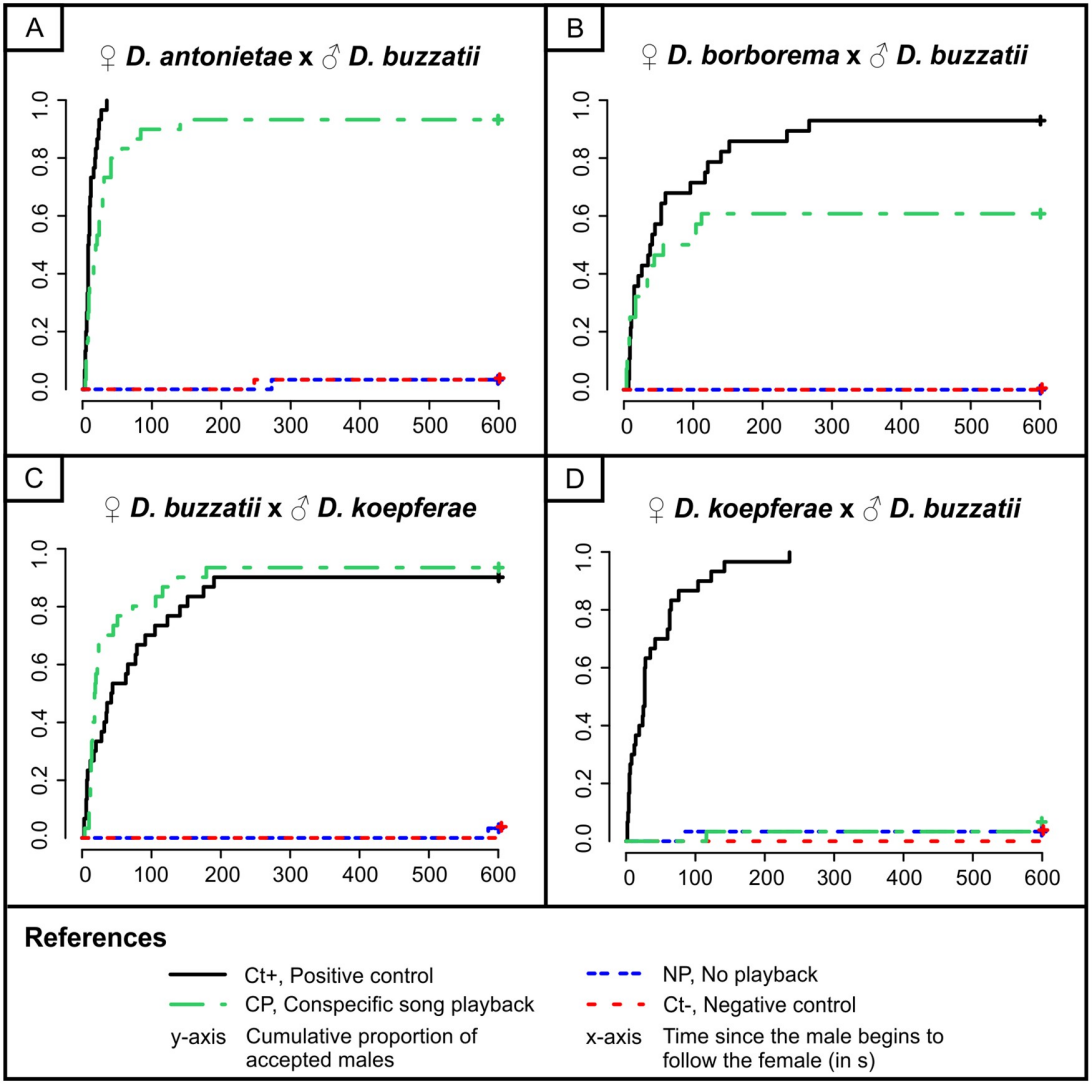
Effect of different acoustic conditions on male acceptance and copulation latency using heterospecific males (PC, NP and Ct-) and conspecific males (Ct+) in Experiment 2. Winged males were used in both controls (Ct+ and Ct-) and wingless males in NP and CP conditions.

In order to assess the significance of courtship song in sexual isolation, we quantified the same response variables (MA and CL) in heterospecific pairs involving females of the *D*. *buzzatii* cluster derived from populations that are sympatric with *D*. *buzzatii* (or with *D*. *koepferae* in the assays involving *D*. *buzzatii* females). Results of Experiment 2 are summarized in Fig 5. All statistical pairwise comparisons and associated adjusted p-values can be found in S3 and S4 Tables for variables MA and CL, respectively.

To demonstrate the existence of precopulatory sexual isolation we exposed females of each species of the *D*. *buzzatii* cluster to winged *D*. *buzzatii* males (or winged *D*. *koepferae* males in assays involving *D*. *buzzatii* females). In all species, MA was completely suppressed in these couples (Ct- condition; Fig 5). Therefore, we can conclude that all heterospecific pairs exhibit strong precopulatory sexual isolation in the time interval considered. Furthermore, MA was also completely abolished when wingless heterospecific males were used with no playback (NP condition; Fig 5). These results suggest that heterospecific song is not responsible for the rejection of winged heterospecific males by females in Ct- condition.

Playing back the conspecific song restored both mate acceptance (CP vs Ct+ conditions, Tukey test, *Z* = 0.46, *P* = 0.97) and copulation latency (CP vs Ct+ conditions, LM: *F*_1,53_ = 0.99, *P* = 0.32) to levels comparable to the Ct+ group in pairs involving *D*. *buzzatii* females and wingless *D*. *koepferae* males (Fig 5C). Therefore, precopulatory sexual isolation is completely eliminated in these pairs by playing back the conspecific song.

Females of *D*. *antonietae* also accepted wingless *D*. *buzzatii* males when conspecific song was played back without significant differences in mate acceptance as compared to the Ct+ group (CP vs Ct+ conditions, Tukey test: *Z* = -0.581, *P* = 0.937). However, there was a significant effect on copulation latency since time to copulation was longer in the CP condition than in the Ct+ group (CP: 27±30 s vs. Ct+: 12±8 s, LM: *F*_1, 56_ = 8.9841, *P* < 0.01). This implies that, although conspecific song playback was sufficient to breakdown precopulatory sexual isolation between *D*. *antonietae* females and wingless *D*. *buzzatii* males, other non-acoustic cues seemed to influence female mate choice by lengthening the time females took to accept wingless heterospecific males.

In trials involving *D*. *borborema* females and wingless *D*. *buzzatii* males, playing back the conspecific song failed to fully restore mate acceptance relative to the Ct+ group [CP: 0.61 vs Ct+: 0.93, *Z* = -2.57, *P* < 0.05(0.0477)]. Nevertheless, more than a half of the females accepted wingless *D*. *buzzatii* males when conspecific song was played back. In this case, although conspecific song playback reduced precopulatory sexual isolation between *D*. *borborema* females and wingless *D*. *buzzatii* males, it was not enough to eliminate it completely.

In contrast, MA was strongly reduced in *D*. *koepferae* regardless of the acoustic condition when females were exposed to *D*. *buzzatii* males (NP, CP and Ct- conditions in Fig 5). The fact that winged and wingless with no playback *D*. *buzzatii* males were not accepted by *D*. *koepferae* females (Fig 5D, Ct- and NP conditions, respectively) suggests that, despite heterospecific song playback has the potential to reduce AM in *D*. *koepferae* (Fig 4D, HP condition), the rejection of wingless *D*. *buzzatii* males would be a consequence of non acoustic cues (Fig 5D, NP condition). In addition, females also did not accept wingless *D*. *buzzatii* males with the conspecific song playback (Fig 5D, CP condition). Therefore, we can conclude that the conspecific song cannot eliminate or reduce precopulatory sexual isolation between *D*. *koepferae* females and *D*. *buzzatii* males.

### Experiment 2: Copulation duration

There was considerable variation in the duration of conspecific copula among *Drosophila* species assessed (Table 1). In heterospecific pairs, the minimum duration of copulation registered was twice the time considered as a criterion to define male acceptance (Table 1). In assays using *D*. *antonietae* females, the average length of heterospecific copulations with conspecific playback (CP) exceeded those of the Ct+ group (Table 1). On the contrary, in trials with females of *D*. *borborema* and *D*. *buzzatii*, the average length of copulation in pairs involving heterospecific males (CP) was shorter than in the Ct+ group (Table 1).

## Discussion

Male courtship songs have undergone a spectacular diversification in the *Drosophila buzzatii* cluster ([24], Fig 3). Accordingly, it has been suggested that sexual selection has played a significant role in promoting rapid diversification, reproductive isolation and speciation [24]. However, there is evidence that the courtship song in *Drosophila* is not always used in female mate choice decisions, as in the case of the Hawaiian *D*. *heteroneura* [15], nor plays the same role when it is taken into account (reviewed in [43] and in [27]). In this vein, our study investigated the role that the courtship song plays in female mate choice and in sexual isolation by evaluating two different aspects: which acoustic conditions favor the acceptance of males (MA) and how fast are they accepted (CL).

### Importance of the courtship song in the *D*. *buzzatii* cluster

Our study revealed that acoustic cues are used by females of the studied species of the *D*. *buzzatii* cluster as a criterion for male acceptance or influence the speed with which males are chosen. Therefore, our results support the hypothesis that sexual selection has played an important role in the diversification of courtship songs in this group of closely related species.

**Fig 6:**
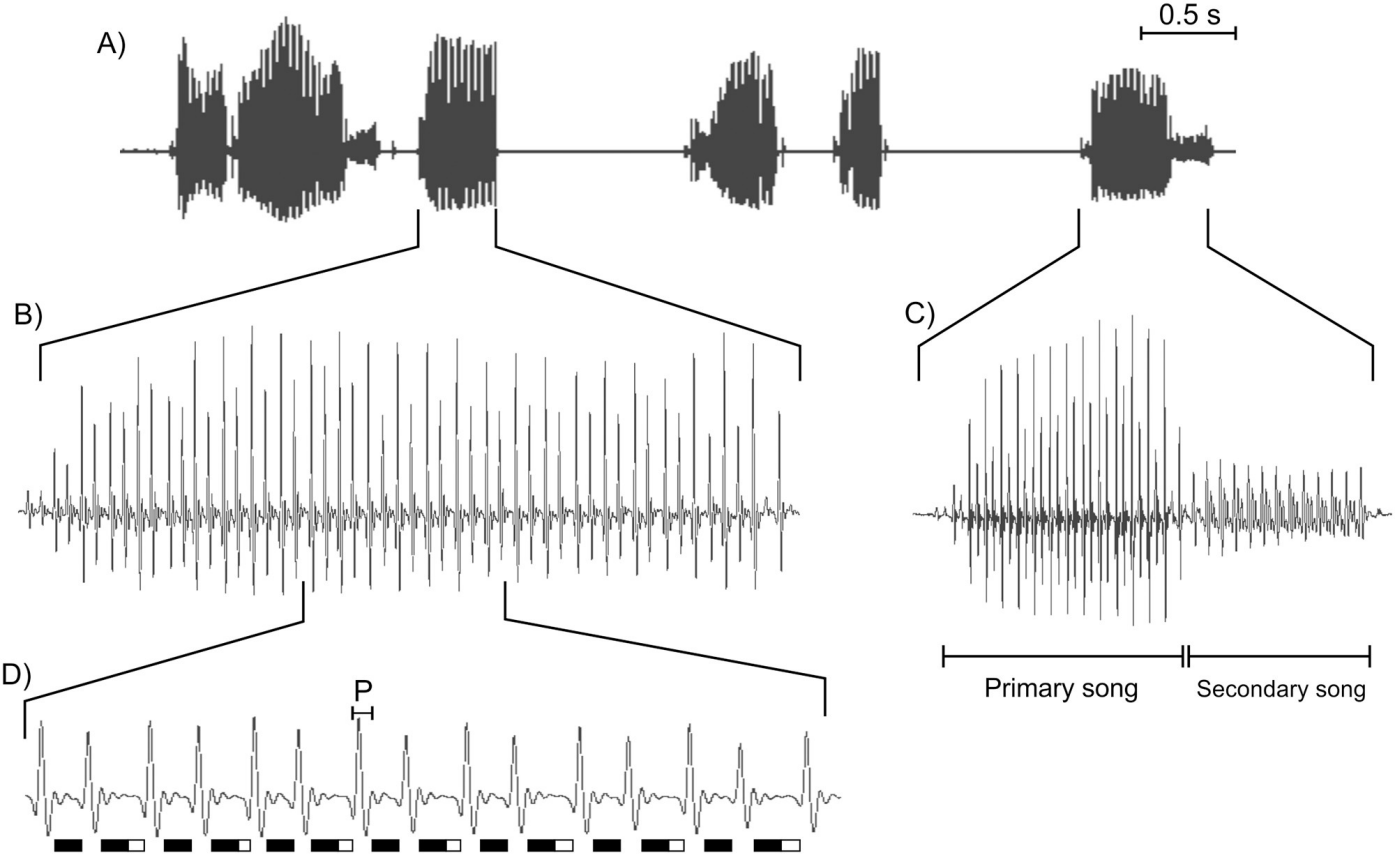
Sonogram of a *D*. *buzzatii* courtship song used in this work illustrating both types of songs and the "doublets of pulses" in the primary song. (A) Six seconds of a *D*. *buzzatii* courtship song recorded for playback experiments. (B) A burst containing primary song alone. (C) A burst containing primary and secondary songs. (D) Expanded view of B illustrating the tendency to alternate pulses with long and short IPIs (doublets of pulses). Black bars indicate the duration of short IPIs, white bars indicate the difference in duration between long and short IPIs and P indicates the pulse duration. This same pattern can be detected in the sonogram of Fig 20C of Oliveira *et al*. [24] by measuring the IPIs.

The courtship song of *D*. *buzzatii* is the most divergent within the *D*. *buzzatii* cluster. It is the only species that has "doublets of pulses" in the primary or A song ([11]; Fig 20C in [24]; P.P. Iglesias pers obs) (Fig 6) and, in addition, the IPIs of these pulses clearly differ from those of the secondary or B song ([24]; P.P. Iglesias pers obs). These particular features of the *D*. *buzzatii* courtship song are an inherent part of the species’ acoustic signal at least in Argentinean and Brazilian populations (Fig 20C in [24]; P.P. Iglesias & E. Hasson unpublished results). Therefore, these characteristics have the potential to make the *D*. *buzzatii* courtship song easily discriminable by females of the remaining species of the cluster and also easily recognizable by conspecific females.

The fact that male acceptance was reduced when wingless conspecific males courted in silence (NP condition) or with the heterospecific song playback (HP condition; Fig 4A–4D) and that the conspecific song playback restored MA and CL to levels observed in the Ct+ group, suggests that there are song parameters that stimulate females in a species-specific manner. In this regard, three song parameters have been reported as cues used by *Drosophila* females for species recognition or sexual selection. IPI is the most widely studied song parameter and has been reported as relevant for species recognition (reviewed in [27]). In addition, studies in *D*. *montana* showed that pulse duration and carrier frequency are also predictors of mating success [44, 45]. In this respect, Oliveira *et al*. [24] found significant overlap for these parameters among species of the *D*. *buzzatii* cluster and suggested that females may use more than one song parameter in mate recognition. Furthermore, it is clear that *D*. *koepferae* females, unlike the other species of the cluster, discriminate conspecific males with the heterospecific song playback more intensely than conspecific males with no playback (Fig 4D; HP and NP conditions).

Taken together, these findings highlight females of *D*. *koepferae* as the most different in terms of the use of the courtship song in mate choice. Females accepted males when the playback was conspecific, intensely reduced male acceptance when playback was heterospecific and exhibited an intermediate response when courtship occurred in the absence of acoustic stimulus (Fig 4D). Since high MA in the absence of playback was accompanied by an increase in CL, the difference in MA between conditions with no playback and with conspecific song playback may be explained by the decrease in the courtship time necessary for males to be accepted by females, which causes an increase in the number of males accepted in the time interval defined in this experiment (*i*.*e*., 10 min). This suggests that *D*. *koepferae* females use other cues (*e*.*g*., hydrocarbons) to discriminate compatible males and song parameters to rank compatible males. Thus, it may be hypothesized that the courtship song alters initial male attractiveness, determined by other cues (as can be seen in the NP condition), and enhances or reduces male mating success as was also reported for *D*. *biauraria* and *D*. *sechellia* [18, 19].

In summary, our results indicate that the importance and the role of conspecific and heterospecific songs differ among species. In *D*. *koepferae*, the evidence suggests that the heterospecific song plays an important role in reducing the number of non compatible matings and that conspecific song is not particularly necessary to achieve copulation, though it reduces the courting time needed for males to be accepted (Fig 4). In the remaining species, the presence or absence of the conspecific song determines male acceptance in most of the females, whereas the heterospecific song does not seem to play a critical role in mate choice because we did not find significant differences between conditions with no playback and with the heterospecific song playback (S1 Table). However, it is important to note that at least one female accepted the conspecific male in the absence of the conspecific song in *D*. *antonietae*, *D*. *borborema* and *D*. *buzzatii*. These observations suggest that there is intraspecific variation among females in relation to the sensory modalities used in mate choice or the degree of female choosiness [4], and that variation is greater in *D*. *antonietae* (Fig 4A–4C). Nevertheless, it is worth mentioning that male acceptance always implied a longer CL in the absence of the conspecific song playback (Fig 4A–4C).

In addition, this study provides evidence that the courtship song emitted either by wing vibration or through a speaker is equivalently processed by the female nervous system. This finding contrasts with results in *D*. *melanogaster*, in which the reproduction of natural or artificially generated conspecific songs consistently reduces MA relative to controls [17, 46–49]. To account for these observations, it has been proposed that wing vibration may also contribute to the spreading of pheromones that would stimulate female receptivity [48]. However, our results show that wings do not provide or facilitate any visual or chemical signal in these species.

### Importance of the courtship song in the *D*. *buzzatii* complex

In order to explore the generality of these results outside the *D*. *buzzatii* cluster, we included *D*. *venezolana* as representative of the closely related *D*. *martensis* cluster. Females of this species accepted conspecific males independently of the acoustic condition (Fig 4E), suggesting that the acoustic stimulus is not relevant during courtship since we did not even find differences in CL. In this sense, our results point towards the idea that other signals influence mate choice in *D*. *venezolana* females.

Despite the fact that we only studied a single species outside the *D*. *buzzatii* cluster, the latter results show that the use of courtship song in female mate choice is not a conserved feature in the *D*. *buzzatii* complex. The study of more species of the *D*. *martensis* cluster is necessary to establish whether the loss of the use of courtship song in female mate choice is a characteristic shared by all or some species of the *D*. *martensis* cluster or it is an autapomorphy of *D*. *venezolana*.

Thus, the evolution of female preferences in the *D*. *buzzatii* complex (composed of the *D*. *buzzatii*, *D*. *martensis* and *D*. *stalkeri* clusters) involved at least one change in the sensory channel used by females to sense males. Females switched from sensing the quality and/or compatibility of males using non-acoustic cues (*e*.*g*., hydrocarbons) to using song parameters, or vice versa. However, to accurately define the branch of the phylogenetic tree in which this evolutionary change arose, and the polarity (direction) of this transformation, it is necessary to study the role of courtship song in female mate choice in more representatives of these clusters and interpret the results in a well sampled phylogenetic hypothesis. In this regard, the *D*. *buzzatii* complex may provide a model system for understanding the reduction or loss of female preferences in species in which the male trait (*i*.*e*., courtship song) is still present [5].

### Courtship song and its role in sexual isolation

*Drosophila buzzatii* stands out as the most divergent species within the *D*. *buzzatii* cluster, not only due to the specific features of its courtship song, but also in ecological, morphological and biogeographical aspects. Given that *D*. *buzzatii* is also the only species that co-occurs with all other species in the cluster (Fig 1), differentiation in courtship songs, and/or in the sensory channels used by females for mate choice, have the potential to reduce or avoid incompatible matings in sympatry. Using heterospecific pairs, we evaluated the role of courtship song in precopulatory sexual isolation between *D*. *buzzatii* and the remaining species of the cluster.

On one hand, we found that females exhibited strong precopulatory sexual isolation when they were exposed to winged heterospecific males (Fig 5A–5D, Ct- acoustic condition) and that isolation was maintained when using heterospecific males without wings, that is, that cannot produce the heterospecific courtship song (Fig 5A–5D, NP condition). Accordingly, we can rule out that the heterospecific song plays a primordial role in sexual isolation. The only species capable of discriminating against the heterospecific song was *D*. *koepferae* (Fig 4, HP condition). However, 40% of females copulated when the *D*. *buzzatii* song was reproduced. Our findings suggest that the ability of most *D*. *koepferae* females to discriminate the heterospecific song when exposed to conspecific males (Fig 4, HP condition) would reinforce precopulatory sexual isolation, but it does not determine it, since isolation was not reduced by eliminating the heterospecific courtship song when using heterospecific males (Fig 5, NP condition).

On the other hand, we found that the importance of the conspecific song in sexual isolation varied among species. Females of *D*. *antonietae*, *D*. *borborema* and *D*. *buzzatii* accepted wingless heterospecific males when we played back the female conspecific song (Fig 5A–5C; CP condition). In contrast, females of *D*. *koepferae* rejected *D*. *buzzatii* males in all acoustic conditions (Fig 5D). However, conspecific song playback did not have the same effect among females of the species that could be deceived and mated with the heterospecific male. Playing back the conspecific song was sufficient to completely break down precopulatory sexual isolation between *D*. *buzzatii* females and *D*. *koepferae* males since no significant differences were found in MA and CL when compared to the Ct+ group (Fig 5C, CP and Ct+ conditions). Courtship song as the main signal responsible for sexual isolation between sympatric species has already been reported in *D*. *montana* [50] and in the *D*. *athabasca* species complex [51]. Nevertheless, our results provide indirect evidence that other cues affect MA and CL in *D*. *antonietae* and *D*. *borborema*. Females of *D*. *antonietae* in the CP condition did not differ from the Ct+ group in MA; however, they took longer to accept *D*. *buzzatii* males than conspecific males (S2 Table; conditions CP and Ct+, respectively). Therefore, we can infer that another type of signal transmitted by *D*. *buzzatii* males through a sensory channel different from the acoustic one is less attractive for *D*. *antonietae* females than those emitted by conspecific males. In *D*. *borborema* assays, on the contrary, MA decreased significantly (Fig 5B; CP condition), yet a large proportion of females were deceived and accepted the heterospecific male. This suggests that other signals are also influencing mate choice in *D*. *borborema*. Sexual signaling in *Drosophila* takes place through visual, auditory and chemosensory cues [6]. Since the species studied in this paper are cryptic [28, 52] and also courtship occurs behind the female (P.P. Iglesias pers obs), males cannot transmit visual information [6]. Thus, we expect that differences in hydrocarbons (volatile and/or not volatile) may explain the observed differences in CL and MA. Hydrocarbons have been shown to play an important role in mate choice in many species of several groups of *Drosophila* such as the *D*. *melanogaster* complex [53], the *D*. *serrata* species group [54], the *D*. *subquinaria* complex [55] and the *D*. *yakuba* species group [56]. However, we cannot rule out the possibility that *D*. *buzzatii* males may differ in their courtship intensity toward *D*. *borborema* females, since *D*. *buzzatii* males not only took longer to start courtship when were exposed to these females (P.P. Iglesias pers obs) but also some males never started to court.

It is important to note that only *D*. *buzzatii* and *D*. *koepferae* lines used in this study come from the same area and, therefore, are truly sympatric populations. On the contrary, females of *D*. *antonietae* and *D*. *borborema* derive from populations sympatric with *D*. *buzzatii*, but allopatric with respect to the *D*. *buzzatii* males used as heterospecifics in this study. There are no previous studies investigating whether courtship song varies among populations in the *D*. *buzzatii* cluster species and whether character displacement (reinforcement) has occurred. However, if character displacement in courtship song has occurred in the areas of sympatry we expect female discrimination against sympatric heterospecific songs to be more drastic than against allopatric ones. Since MA was almost completely reduced in the assays with *D*. *antonietae* and *D*. *borborema* females (Fig 3, HP condition) we do not expect our broad conclusions to be substantially altered by the use of sympatric *D*. *buzzatii* males in Experiment 1. However, the use of sympatric *D*. *buzzatii* males in Experiment 2 might reduce the effect of the conspecific song playback in the assays with *D*. *antonietae* and *D*. *borborema* females if there is character displacement in signals perceived by sensory modalities other than acoustics. Nevertheless, our results from Experiment 1 suggest that non acoustic cues would not be as important for *D*. *antonietae* and *D*. *borborema* females as they are for *D*. *koepferae* females given the strong reduction in MA observed in assays with *D*. *antonietae* and *D*. *borborema* females in the no playback condition relative to what was observed in *D*. *koepferae* (Fig 4A, 4B and 4D; NP condition).

### Copulation duration

To determine the acceptance of the male in heterospecific pairs of Experiment 2, we used the criterion of considering as acceptance the cases in which the pair remained in the copulation position and the female did no exhibit rejection behavior for at least 10 s. Since *D*. *buzzatii* has the most divergent morphology of the intromittent organ (aedeagus) in comparison to the other species of the cluster [28], one could speculate that the ability to remain in copulation position could be prevented in heterospecific pairs by some kind of mechanical incompatibility as predicted by the key and lock hypothesis [57]. However, according to this hypothesis, reproductive isolation could operate through two different mechanisms (reviewed in [58]). The first mechanism is called classical or structural lock-and-key and corresponds to the existence of mechanical incompatibilities that reduce or prevent copulation and/or insemination [59, 60]. The second mechanism is called sensory lock-and-key and proposes that morphological differences are perceived by one or both sexes and evoke behavioral or physiological responses that result in the premature termination of copulation or a reduction of postcopulatory reproductive fitness [61]. An increasing body of experimental evidence does not support the structural mechanism. However, it does not rule out that the sensory mechanism can be widely extended, limiting gene flow among species (reviewed in [58]).

Our results show that there were significant differences in MA among acoustic conditions considering the 10 s time interval in the assays involving *D*. *antonietae*, *D*. *borborema* and *D*. *buzzatii* females, suggesting that the ability to remain in copulation position is not prevented in these heterospecific pairs (Fig 5). Only in the assays involving *D*. *koepferae* females we did not observe pairs in copula (Fig 5). The aedeagus size and shape of *D*. *koepferae* males is similar to that of *D*. *antonietae* males [28]. However, all *D*. *antonietae* females did copulate with wingless *D*. *buzzatii* males in the CP condition. In fact, when *D*. *antonietae* females were used, the minimum time of permanence in copulation position was 89 s (Table 1) and the mean time of copulation duration was even higher than in the corresponding Ct + pairs (Table 1). This provides indirect evidence that the divergent size and shape of the aedeagus of *D*. *buzzatii* may not be responsible for the results found in *D*. *koepferae* trials. The lowest time in copula was recorded in heterospecific pairs involving *D*. *borborema* females, even though males of *D*. *borborema* have intermediate aedeagus morphology between the aedeagus of *D*. *buzzatii* and those of *D*. *antonietae* and *D*. *koepferae* [28]. Nevertheless, the minimum time recorded doubled the time used as acceptance criterion. These results suggest that differences in aedeagus size and shape are not fair predictors of both MA and duration of copulation in the heterospecific pairs assessed.

Our findings do not support the mechanical lock and key mechanism hypothesis but do not contradict the sensory lock-and-key mechanism expectations. Therefore, these results indicate that the criterion used to define male acceptance in heterospecific pairs of Experiment 2 is appropriate to avoid confusing the effect of precopulatory (*e*.*g*., courtship songs) and postcopulatory (*e*.*g*., aedeagus morphology) traits in female mate choice.

## Conclusions

Our study revealed that acoustic cues are used by females of the studied species of the *D*. *buzzatii* cluster as a criterion for male acceptance or influence the time females take to accept the male. Since our study only included four species of the *D*. *buzzatii* cluster (*D*. *antonietae*, *D*. *borborema*, *D*. *buzzatii* and *D*. *koepferae*), we cannot ensure that our observations can be extended to the other three species of the cluster (*i*.*e*., *D*. *gouveai*, *D*. *serido* and *D*. *seriema*). Furthermore, the fact that this characteristic is not shared by *D*. *venezolana*, a species of the *D*. *martensis* cluster that we use as external group, indicates that there is a higher diversity in the sensory modalities used by females during courtship in the *D*. *buzzatii* complex. We also found that the studied species of the *D*. *buzzatii* cluster differ in the role that conspecific and heterospecific songs have in female mate choice and in sexual isolation in relation to the only semi-cosmopolitan species, *D*. *buzzatii*. Thus, our findings indicate that divergence in female preferences for courtship songs and the differential use of alternative sensory modalities during courtship are drivers of acoustic divergence in the *D*. *buzzatii* cluster. In this sense, the results suggest that courtship songs may have played a predominant role in the evolution of this species cluster as it successfully diversified in the xeric areas of South America. However, more comprehensive studies would help to shed light on whether these particular signals were more important early (when there is little genetic and no morphological divergence) or later (during secondary contact in sympatry) in the speciation process. The well-known ecology of some of these species provides the opportunity to disentangle the relationship among courtship songs, hydrocarbons and host cacti in the evolution of sexual isolation in a group of species with varying divergence times.

Finally, our study also revealed intraspecific variation in female preferences or female choosiness since a small number of females behaved differently in some acoustic conditions. Therefore, future studies should focus on investigating the causes that underlie variation in female behavior and on studying patterns of intraspecific variation in each species.

## Supporting information

S1 Table Pairwise comparisons between different acoustic conditions for the response variable Mate Acceptance (MA) of Experiment 1. (DOCX)

S2 Table Pairwise comparisons between different acoustic conditions with more than 50% of Mate Acceptance (MA) for the response variable Copulation Latency (CL) of Experiment 1. (DOCX)

S3 Table Pairwise comparisons between different acoustic conditions for the response variable Mate Acceptance (MA) of Experiment 2. (DOCX)

S4 Table Pairwise comparisons between different acoustic conditions with more than 50% of Mate Acceptance (MA) for the response variable Copulation Latency (CL) of Experiment 2. (DOCX)

S1 Appendix Raw data from Experiment 1. (XLSX)

S2 Appendix Raw data from Experiment 2. (XLSX)
