# Supplementary material for: The role of courtship song in female mate choice in South American Cactophilic Drosophila
Source: PLoS One. 2017 May 3;12(5):e0176119. doi: 10.1371/journal.pone.0176119 (PMC5414974; doi:10.1371/journal.pone.0176119)
Supplement: S1 Table — (DOCX) [file pone.0176119.s001.docx]

**S1 Table**

Pairwise comparisons between different acoustic conditions for the response variable mate acceptance (MA) of Experiment 1.

|  | ♀ *D. buzzatii* | | | ♀ *D. koepferae* | | ♀ *D. antonietae* | | ♀ *D. borborema* | | | ♀ *D. venezolana* | | |  |
| --- | --- | --- | --- | --- | --- | --- | --- | --- | --- | --- | --- | --- | --- | --- |
|  | *Z* | *P* | *Z* | | *P* | *Z* | *P* | | *Z* | *P* | | *Z* | *P* | |
| CP – Ct+ | 0.578 | 0.933 | 0.310 | | 0.989 | 1.054 | 0.698 | | -0.581 | 0.937 | | – | NS† | |
| HP – NP | 1.714 | 0.293 | 4.773 | | **<0.001** | 1.762 | 0.271 | | -0.581 | 0.937 | | – | NS† | |
| Ct+ – NP | -4.973 | **<0.001** | -3.547 | | **<0.01** | -5.420 | **<0.001** | | -4.682 | **<0.001** | | – | NS† | |
| HP – CP | -5.323 | **<0.001** | 6.604 | | **<0.001** | -4.804 | **<0.001** | | 5.099 | **<0.001** | | – | NS† | |
| NP – CP | -4.961 | **<0.001** | -3.691 | | **<0.01** | -4.448 | **<0.001** | | -4.793 | **<0.001** | | – | NS† | |
| Ct+ – HP | -5.316 | **<0.001** | -6.681 | | **<0.001** | -6.592 | **<0.001** | | -4.793 | **<0.001** | | – | NS† | |

CP = Conspecific playback, Ct+ = Positive control, HP = Heterospecific playback, NP = No playback, NS = Non significant.

† No pairwise comparison was made because GLM analysis was not significant: $\chi_{3}^{^{2}}$= 1.660; *P* = 0.646.

See Materials and Methods section for details on statistical analysis and acoustic conditions.
