# Supplementary material for: The role of courtship song in female mate choice in South American Cactophilic Drosophila
Source: PLoS One. 2017 May 3;12(5):e0176119. doi: 10.1371/journal.pone.0176119 (PMC5414974; doi:10.1371/journal.pone.0176119)
Supplement: S2 Table — (DOCX) [file pone.0176119.s002.docx]

**S2 Table**

Pairwise comparisons between different acoustic conditions with more than 50% of mate acceptance (MA) for the response variable copulation latency (CL) of Experiment 1.

|  | ♀ *D. buzzatii* | | | ♀ *D. koepferae* | | | ♀ *D. antonietae* | | | ♀ *D. borborema* | | | ♀ *D. venezolana* | | |
| --- | --- | --- | --- | --- | --- | --- | --- | --- | --- | --- | --- | --- | --- | --- | --- |
|  | *Z* | *F** | *P* | *Z* | *F** | *P* | *Z* | *F** | *P* | *Z* | *F* | *P* | *Z* | *F* | *P* |
|  |  | (df) |  |  | (df) |  |  | (df) |  |  | (df) |  |  | (df) |  |
| CP – Ct+ | – | 0.610 | 0.49 | 1.92 | – | 0.128 | – | 5.376 | 0.10 | – | 0.172 | 0.68 | – | – | NS† |
|  |  | (1,2.99) |  |  |  |  |  | (1,2.99) |  |  | (1,56) |  |  |  |  |
| HP – NP | – | – | – | – | – | – | – | – | – | – | – | – | – | – | NS† |
| Ct +– NP | – | – | – | 6.77 | – | **<0.001** | – | – | – | – | – | – | – | – | NS† |
| HP – CP | – | – | – | – | – | — | – | – | – | – | – | – | – | – | NS† |
| NP – CP | – | – | – | 4.93 | – | **<0.01** | – | – | – | – | – | – | – | – | NS† |
| Ct+ – HP | – | – | – | – | – | – | – | – | – | – | – | – | – | – | NS† |

CP = Conspecific playback, Ct+ = Positive control, HP = Heterospecific playback, NP = No playback, df = degrees of freedom, NS = Non significant.

* F tests were based on Kenward-Roger's adjusted degrees of freedom solution (mixed models).

† No pairwise comparison was made because LM analysis was not significant: *F*_3,102_ = 0.457; *P* = 0.713.

See Materials and Methods section for details on statistical analysis and acoustic conditions.
