# Supplementary material for: The role of courtship song in female mate choice in South American Cactophilic Drosophila
Source: PLoS One. 2017 May 3;12(5):e0176119. doi: 10.1371/journal.pone.0176119 (PMC5414974; doi:10.1371/journal.pone.0176119)
Supplement: S3 Table — (DOCX) [file pone.0176119.s003.docx]

**S3 Table**

Pairwise comparisons between different acoustic conditions for the response variable mate acceptance (MA) of Experiment 2.

|  | ♀ *D. buzzatii* | | | ♀ *D. koepferae* | | | ♀ *D. antonietae* | | | ♀ *D. borborema* | | |
| --- | --- | --- | --- | --- | --- | --- | --- | --- | --- | --- | --- | --- |
|  | *Z* |  | *P* | *Z* |  | *P* | *Z* |  | *P* | *Z* |  | *P* |
| PC – Ct- | 5.099 |  | **<0.001** | 0.581 |  | 0.937 | 5.099 |  | **<0.001** | 3.425 |  | **<0.01** |
| Ct+ – NP | -4.695 |  | **<0.001** | -4.793 |  | **<0.001** | -4.793 |  | **<0.001** | -4.669 |  | **<0.001** |
| Ct- – NP | -0.581 |  | 0.937 | 0.581 |  | 0.937 | 0.000 |  | 1.000 | 0.000 |  | 1.000 |
| Ct+ – PC | 0.464 |  | 0.966 | -4.793 |  | **<0.001** | -0.581 |  | 0.937 | -2.567 |  | **<0.05** |
| NP – PC | -4.793 |  | **<0.001** | 0.000 |  | 1.000 | -5.099 |  | **<0.001** | -3.425 |  | **<0.01** |
| Ct- – Ct+ | -5.081 |  | **<0.001** | -4.682 |  | **<0.001** | -4.793 |  | **<0.001** | -4.669 |  | **<0.001** |

CP = Conspecific playback, Ct- = Negative control (using winged heterospecific males), Ct+ = Positive control (using winged conspecific males), NP = No playback.

See Materials and Methods section for details on statistical analysis and acoustic conditions.
