# Supplementary material for: The role of courtship song in female mate choice in South American Cactophilic Drosophila
Source: PLoS One. 2017 May 3;12(5):e0176119. doi: 10.1371/journal.pone.0176119 (PMC5414974; doi:10.1371/journal.pone.0176119)
Supplement: S4 Table — (DOCX) [file pone.0176119.s004.docx]

**S4 Table**

Pairwise comparisons between different acoustic conditions with more than 50% of mate acceptance (MA) for the response variable copulation latency (CL) of Experiment 2.

|  | ♀ *D. buzzatii* | | | ♀ *D. koepferae* | | | ♀ *D. antonietae* | | | ♀ *D. borborema* | | |
| --- | --- | --- | --- | --- | --- | --- | --- | --- | --- | --- | --- | --- |
|  |  | *F* | *P* |  | *F* | *P* |  | *F* | *P* |  | *F* | *P* |
|  |  | (df) |  |  | (df) |  |  | (df) |  |  | (df) |  |
| CP – Ct- |  | – | – |  | – | – |  | – | – |  | – | – |
| Ct+ – NP |  | – | – |  | – | – |  | – | – |  | – | – |
| Ct- – NP |  | – | – |  | – | – |  | – | – |  | – | – |
| Ct+ – PC |  | 0.991 | 0.324 |  | – | – |  | 8.984 | **<0,01** |  | 2.169 | 0.148 |
|  |  | (1,53) |  |  |  |  |  | (1,56) |  |  | (1,41) |  |
| NP – PC |  | – | – |  | – | – |  | – | – |  | – | – |
| Ct- – Ct+ |  | – | – |  | – | – |  | – | – |  | – | – |

CP = Conspecific playback, Ct- = Negative control (using winged heterospecific males), Ct+ = Positive control (using winged conspecific males), NP = No playback, df = degrees of freedom.

See Materials and Methods section for details on statistical analysis and acoustic conditions.
